# Psychoneuroimmunoendocrinology: clinical implications

**DOI:** 10.1186/s40413-017-0151-6

**Published:** 2017-06-06

**Authors:** Sandra Nora González-Díaz, Alfredo Arias-Cruz, Bárbara Elizondo-Villarreal, Olga Patricia Monge-Ortega

**Affiliations:** Department of Allergy and Clinical Immunology Service, University Hospital “Dr. José Eleuterio González” Autonomous University of Nuevo Leon (UANL), Monterrey, Nuevo León 64460 Mexico

**Keywords:** Psychoneuroimmunoendocrinology, Stress, Immune system, Endocrine system, Nervous system, Psychiatry, Allergy, Behavior, Psychological disorders, Immunology

## Abstract

Psychoneuroimmunoendocrinology, which was first described in 1936, is the study of the interactions between the psyche, neural and endocrine functions and immune responses. The aim of psychoneuroimmunoendocrinology is to apply medical knowledge to the treatment of different allergic, immune, autoimmune, rheumatic, neoplastic, endocrine, cardiovascular and dental pathologies, among other disorders. Epigenetic factors and major stresses from different types of stimuli acting through distinct pathways and neurotransmitters are highly involved in altering the psychoneuroimmunoendocrine axis, resulting in the emergence of disease. The main purpose of this report is to expand the understanding of psychoneuroimmunoendocrinology and to demonstrate the importance of the above-mentioned interactions in the etiology of multiple pathologies. In this review, a search of the medical literature using PubMed (free access search engine for the Medline database of the National Library of Medicine of the United States) over the years 1936 to 2016 was conducted, and descriptive and experimental studies and reviews of the scientific literature were included.

## Background

Each field of medicine has a defined scope; however, interactions between systems covered by different fields exist. A link between findings from psychiatry, neurology, immunology and endocrinology has been noted for many years. From the functional, anatomical and physiological perspectives, the correlation between the central nervous system (CNS) and the endocrine system is complex and involves several actors, such as cytokines, receptors and neurotransmitters. The immune system is connected to the endocrine and neural systems via a number of pathways that integrate the functions of the hypothalamus, pituitary glands, adrenal glands, thyroid glands, gonads and autonomic nervous system. Major clinical implications and a vast amount of pathologies are related to the relationships between the systems covered by the science of psychoneuroimmunoendocrinology.

Many studies have described the negative effects of stress on health. Ader and Cohen in 1975 studied the effects of stress on the immune system. These previous findings are currently accepted, and a new area of study focusing on inflammation, autoimmunity and secondary hypersensitivity to stress has been developed.

Neuroimmunophysiologists have found that stress, anxiety and depression not only affect the protective function of the immune system but also alter its regulatory function.

Socioeconomic status as well as chronic illnesses such as autoimmune and rheumatic diseases, asthma, allergic rhinitis, atopic dermatitis, urticaria, cardiovascular disease, hypertension and diabetes mellitus affect mood by generating stress, anxiety and depression, all of which negatively influence immune system function and regulation. For example, asthma was historically referred to as “nervous asthma” in relation to living with a histrionic mother, and atopic dermatitis was referred to as “neurodermatitis.”

This review seeks to gather knowledge and the main clinical implications from the field of psychoneuroimmunoendocrinology.

## Psychoneuroimmunoendocrinology

Psychoneuroimmunoendocrinology is the study of the interaction between the psyche, neural function, endocrine function and immune responses. These systems can interact through two pathways: changes in neural and endocrine functions that alter immune responses or stimulation of immune responses that modify the functionality of the endocrine system and the CNS. Behavioral processes are able to initiate both pathways, which leads to altered behavior in an individual [1]. Interactions between these various systems regulate a variety of physiological processes, and their normal interaction helps to reduce the vulnerability of individuals to certain diseases [2, 3]. One aim of psychoneuroimmunoendocrinology is to apply medical knowledge to different psychological disorders (e.g., depression), neurological conditions (e.g., dementia), immune disorders (e.g., autoimmune diseases) and neoplastic diseases [4].

## Background and origin of psychoneuroimmunoendocrinology

The theory of “general adaptation syndrome” proposed in 1936 relates stress with cortico-adrenal secretion and accounts for the protective effects of adrenal extracts on stress [5]. Subsequently, Solomon et al., introduced a “speculative theory”, later known as psychoimmunology, and following the introduction of this theory, Ader and Cohen published their work on classical conditioning of immune functions entitled “Behavioral conditioning of immunosuppression”, in which they proposed a functional link between the immune system and the nervous system such that the immune response generates cytokines that stimulate the CNS [6, 7]. This stimulation of the brain activates the hypothalamic-pituitary-adrenal axis, which in turn suppresses the immune response through the secretion of glucocorticoids [8]. Accumulating evidence since 1980 has established the mechanism by which thoughts, emotions and behavior modulate and mediate endocrine and immune functions [7].

## Pathophysiology of the psychoneuroendocrine axis

The presence of cellular receptors in the nervous, endocrine and immune systems allows the reception of information from other systems via chemical messengers. Under normal conditions, these three systems interact to establish a homeostatic balance [4, 9] that promotes adequate health and prepares the body for constant struggle against various diseases. The loss of this balance represents an interruption in the processes of interaction among these four systems, resulting in the onset of symptoms that characterize a pathogenic state. Many factors, such as heredity, environment, personality traits, emotions and lifestyles, influence these interactions. Whether the stress generated by psychiatric disorders such as depression and anxiety, behavior disorders, daily hassles, and changes in the environment helps or impairs the control of chronic inflammatory diseases remains under debate. It is common to observe people who thrive in environments or situations of high stress as well as people whose health is negatively affected by such stress [7].

Although no specific atopic personality profile has been established, growing knowledge of the nervous system supports new findings concerning the interaction between neuroimmunological and epigenetic factors. The skin and nervous system share a common origin: the ectoderm. Any factor that plays a neurological role can be observed in keratinocytes, fibroblasts, wandering or resident cells (mast cells, Langerhans cells), or stem or transient cells (lymphocytes, neutrophils and monocytes) of the skin. Thus, primitive defense responses such as inflammation and stress may modulate endocrine, dermatological and neurological responses [9, 10].

Stress modulates the immune response through the psychoneuroimmunoendocrine pathway and the hypothalamic-pituitary-adrenal axis [11] via the release of cortisol, norepinephrine, epinephrine and interferon-gamma (IFN-γ) by T lymphocytes. Increased levels of proinflammatory cytokines such as IFN-γ (T helper cell type 1 (Th1) cytokine) and a rapid but tissue-damaging cellular immune response constitute the immune system response [12]. Cortisol and catecholamines decrease the production of tumor necrosis factor-alpha (TNF-α) by antigen-presenting cells and promote Th2 responses via the release of interleukin (IL)10, IL13 and IL4 [13]. This process permits the immune system to halt acute responses but also favors allergic diseases [14, 15]. It has recently been found that epigenetic factors encourage the development of inadequate stress responses, paving the way for a chronic stress response instead of an acute stress response [10, 16, 17].

Table 1 shows a summary of different studies providing scientific relevance regarding the effect of stress on the immune system.

**Table 1:** Relevant studies about the influence of psychological stress on the immune system

References	Main conclusions
Ortega M. 2006 [90]	Stress is a risk factor for health in all systems of the body; even though a certain level of stress is essential to boost productivity, once a limit is exceeded by an intense stressor, the body becomes depleted, causing stress-associated diseases.
Rosenthal S. 2002 [91] Ray O. 2004 [92] Sierra R et al. 2006 [93] Sandín B. 2008 [94] McEwen B.S. 2008 [95]	An altered immune system caused by stressful events increases the body’s vulnerability (to infectious diseases, cancer and autoimmune diseases).
Borysenko J, Borysenko M. 1983 [96] Gidron Y et al. 2003 [97] Ho W, Evans D, Douglas S. 2002 [98]	Social, physical, and biological factors that cause stress may induce immunosuppression, including inability to adapt to the environment, trauma, major surgical interventions, radiation, infection, and cancer.
Monjan A, Collector M. 1977 [99]	Exposure to chronic stress induced proliferation of T and B lymphocytes similar to or to an even greater than a control treatment. Additionally, exposure of mice to acute noise stress for more than 2 to 3 h a day for fewer than 2 consecutive days reduced B and T lymphocyte proliferation in response to the mitogens lipopolysaccharide and concanavalin A, compared to non-exposure to stress.
Fillion L et al. 1994 [100] Weiss J et al. 1989 [101] Hucklebridge F, Clow A, Evans P. 1998 [102]	Stress is related to increased viral retention in tissues, along with a decrease in the number of circulating lymphocytes and in the mitogenic response in vitro. In addition, stress diminishes the activity of natural killer (NK) cells, a powerful mechanism for the elimination of tumor cells and the production of interferon-γ.
Moynihan J, Ader R, Crota L et al. 1990 [103]	Most immune responses are suppressed by stress, but moderately intense stress conditions can increase them.
Croset G et al. 1987 [104]	Rat immune system reactivity was tested by determining the proliferative response after mitogenic stimulation in vitro as well as the capacity to generate a primary antibody response after immunization with red blood cells from sheep. An increase in the immune response in vitro and in vivo was demonstrated following exposure to a single shock. Thus, it was concluded that emotional stimuli facilitate immune responses. However, when a rat was confronted with a conflict situation, there was a decrease in the reactivity of the immune system. These findings led to the final conclusion that the immune system specifically and immediately reacts to different psychological stimuli.
Shirinsky I, Shirinsky V. 2001 [105] Belova T et al. 1988 [106]	Immune reaction initiation may be strongly affected by stress-induced cerebrovascular damage.
Churin A et al. 2003 [107]	Immobilization stress induces different immune system reactions in distinct strains of rodents. These reactions can be classified according to the intensity of the humoral immune response for thymus-dependent antigens into categories such as high, moderate and low responders. High and moderate responders are characterized by high sensitivity of the productive phase of the humoral immune response and the phagocytic activity of macrophages. In low responders, stress only slightly affected the productive phase of the humoral immune response, but peritoneal macrophage activity decreased. This evidence reflects the different reactions of the immune system.
De Groot J et al. 2002 [108]	This study of stress revealed the consequences of stress on the quality and quantity of immunological memory in the long term. Mice were subjected to social stress after herpes simplex virus infection. Stressed mice were shown to exhibit suppressed antibody response and impaired memory for the production of IL4 and IL10 as a specific response to the virus, whereas non-stressed mice showed intact immune responses and immune memory.
Guayerbas N et al. 2002 [109]	This study found that on standard behavioral tests, rodents with high levels of anxiety had less competition in their immune system (premature immunosenescence), as demonstrated by certain functional alterations of peritoneal macrophages, such as substrate adherence, chemotaxis, phagocytosis, and superoxide anion production.
Zelena D et al. 2003 [110]	In rats subjected to stress by repeated trapping, chronic stress signs including decreased thymus size and weight, increased adrenal gland weight, and increased basal corticosterone levels were observed.
Molina P. 2001 [111]	Studies in rats subjected to hemorrhagic shock stress showed a suppressive role of noradrenergic innervation in the increase in tissue TNF-α levels initiated by hemorrhage in vivo. Therefore, it was concluded that norepinephrine protects against tissue damage but may contribute to generalized immunosuppression following trauma.
Wonnacott K, Bonneau R. 2002 [112]	In a murine model, stress reduced the ability of specific cytotoxic memory T lymphocytes to protect against lethal intranasal or intravaginal infection with a herpes simplex virus. Stress also restricted the ability of these lymphocytes to limit virus levels at the site of the infected mucosa.
Paltrinieri S et al. 2002 [113]	The efficiency of granulocytes was studied in sheep subjected to acute stress, and the results demonstrated that acute stress significantly increased the adhesion of these cells. This mechanism could be responsible for the depression of innate immunity observed in stressed animals.
Sánchez M, Cruz C. 1991 [114]	Human studies revealed that IgA class antibodies, which are important in the defense against viruses and bacteria, had reduced abundance in individuals with a particular personality type.
Stowell J. 2003 [115]	In humans, certain academic examinations can have a noteworthy impact on mental and physical health.
Matalka K. 2003 [116]	A review of mental stress models (short and written examinations as subacute and acute types of stressors) was conducted to understand the effects of stress on the neuroendocrine and immune systems. In stressed students, a short period (minutes) of preparation for a written exam induced the production of proinflammatory cytokines, which could be related to a Th1 response. Nevertheless, prolonged mental stress (of several days) caused deregulation of immune function, with a change in the cytokine response to a Th2 response.
Anyanwu E et al. 2003 [117]	Abnormal NK cell activity was found in patients exposed to toxigenic materials, leading to adverse health conditions, including a wide range of neuroimmune and behavioral consequences.
Ho C et al. 2001 [118]	Measurable changes in dendritic cell abundance were observed in patients undergoing surgery. These cells were rapidly mobilized in the circulation in response to surgical stress, and this activity may prepare host immune defenses against trauma.
Woiciechowsky C et al. 1998 [119]	In patients with sympathetic activation due to acute accidental brain trauma, rapid systemic release of the anti-inflammatory cytokine IL10 from non-stimulated monocytes occurs. The rapid release of this cytokine may signify a common pathway for stress-induced immunosuppression.
Dhabhar F, McEwen B. 1999 [120]	Divergent from the concept that stress impairs immunity, human studies showed that short-term stressors pointedly increase delayed hypersensitivity reactions of skin.
Glaser R. 2005 [121]	Individuals exposed to chronic diseases are more likely to present deleterious health and hygiene habits compared to individuals who do not have stress, such as sleep disturbances, malnutrition, physical inactivity and drug and tobacco abuse; thus enhancing the adverse effects of stress on the immune system and overall health.
Levitina E. 2001 [122]	Immunological studies in infants who suffered from perinatal hypoxic stress demonstrated impaired cellular immunity (lymphocyte subpopulations) and humoral immunity (immunoglobulin concentrations). Acute hypoxia led to transient immunodeficiency due to stress.
Ramos, V et al. 2008 [123]	Chronic and excessive stimulation of the hypothalamic-pituitary-adrenal axis induces the production of glucocorticoids, the final products of this axis, altering the levels of white blood cells, decreasing the activity of NK cells and inhibiting the production and secretion of ILs that are important in mediating the immune response.
Mohr D, Pelletier D. 2004 [124]	Stress in individuals with multiple sclerosis increases the permeability of the blood–brain barrier to immune cells circulating in the blood. As a result, there is an increase in the infiltration of leukocytes into the CNS.
Selye H. 1936 [125]	Hypotrophy of the thymus and lymph nodes was demonstrated after exposure to stress. The immunomodulatory effect of glucocorticoids is essential to this effect.
Kay G et al. 1998 [126]	Prenatal stress from maternal isolation and exposure to noise and intense light during the last week of gestation in rats reduced the proliferative response of B lymphocytes and decreased the cytotoxic activity of NK cells in peripheral blood.
Spitzer et al. 2010 [127]	People diagnosed with post-traumatic stress disorder were found to be significantly more likely to have elevated C-reactive protein levels.
Gill J, Page G 2008 [128] Gola et al. 2013 [129] Sutherland, A., Alexander, D. Hutchison, J.2003 [130] von Kanel et al. 2006 [131] von Kanel et al. 2007 [132] Baker et al. 2001 [133] Maes et al. 1999 [134] Newport D, Nemeroff C. 2000 [135]	Research showed that people with post-traumatic stress disorder have elevated levels of proinflammatory cytokines, especially IL6, which has been considered a potential prognostic biomarker for this pathology.
Gotovac et al. 2010 [136] Pace et al. 2012 [137]	Evidence was found for cytotoxic changes to NK cells in people with post-traumatic stress disorder, as well as an increase in the number of glucocorticoid receptors on lymphocytes and a decrease in the sensitivity to glucocorticoids.
Cohen et al. 2001 [138] Herbert T, Cohen S. 1993 [139]	Reduced NK cell cytotoxicity, suppressed lymphocyte proliferative responses, and blunted humoral responses to immunization were found in chronic stress models.
Montoro J et al. 2009 [140]	Activation of the neuroendocrine and sympathetic nervous systems through catecholamine and cortisol secretion influences the immune system, modifying the balance between Th1 and Th2 responses in favor of the Th2 response.

## Stress and the psychoneuroimmunoendocrine axis

In 1936, the concept of general adaptation syndrome and its phases of alarm, resistance and exhaustion in response to an aggression were first proposed [5]. Currently, consensus continues to be sought concerning the definition of stress, for which terms such as homeostatic imbalance, a discrepancy between expectations and perceptions of the environment, and allostasis are used. Allostasis is the ability to maintain a stable internal environment despite the influence of external elements, i.e., adaptation. Adaptation is not achieved when the response is ineffective or inadequate or when exposure to the agent that induces the response is prolonged, resulting in allostatic load, which is defined as wear and tear from the under- or overactivity of allostatic systems [18]. Allostatic load is increased by an overreaction of the adaptive mechanisms capable of generating a disease, transforming a protective mechanism that maintains systemic homeostasis when faced with an aggression into a highly pathogenic mechanism with a prolonged effect. Stress is defined as a real or interpreted threat to the physiological or psychological integrity of an individual that results in specific, physiological or behavioral responses seeking to restore homeostasis and whose chronicity is potentially pathogenic [19]. Castrillón et al. defined psychological stress as a pathophysiological process that occurs when an individual is faced with environmental demands that exceed his or her resources, inducing a response that involves physiological and cognitive activation of the body (CNS, endocrine system and immune system) in order to quickly and forcefully meet the demands of the situation. Therefore, the response to psychological stress is systemic in nature and has several metabolic consequences, such as increased steroid synthesis and a state of chronic inflammation [1]. The response of the body to stress involves the participation of different homeostatic regulatory systems, causing functional alterations that lead to chronic stress, which forms the basis for the development of cardiovascular, metabolic, immunologic, allergic, oncologic and psychiatric disease. An individual’s response to stress is provoked by genetic and psychological factors, which explains the large interindividual variability in the response to similar stimuli [18]. Different stressors cause distinct responses through the activation of specific neuroendocrine systems [19].

**Fig. 1:**
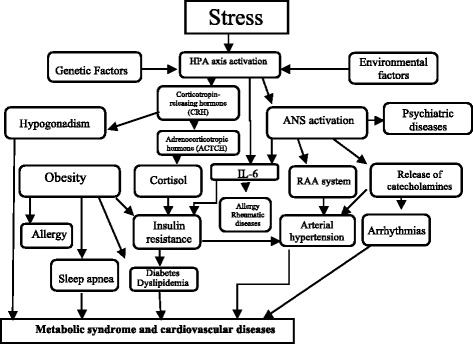
Stress and psychoneuroimmunoendocrinology axis. ANS: autonomic nervous system; HPA: hypothalamic-pituitary-adrenal; IL-6: interleukin 6; RAA: renin-angiotensin-aldosterone

The following points are taken into account when explaining pathophysiological stress: first, the emotional, behavioral and physiological components of a stress reaction are controlled by corticotropin-releasing hormone; second, the intensity and duration of the reaction of the hypothalamic-pituitary-adrenal axis to stress are modulated by the release of glucocorticoids from the hippocampus, which is very sensitive to hippocampal neuronal activity and glucocorticoid insufficiency, and variation in the effectiveness of the brake system for hypothalamic-pituitary-adrenal axis activity likely accounts for interindividual differences in stress responses; and third, through a combination of cytokines and glucocorticoids, the reciprocal interactions between the immune system and the CNS constitute another regulatory element, and altered function of these interactions can be the origin of a pathology [20]. Chronic stress produces alterations in hippocampal neurons, resulting in memory problems. Similarly, chronic stress can suppress immune system defenses and produce a range of psychophysiological symptoms such as adrenal fatigue caused by reduced cortisol levels. Emotional distress has a direct influence on inflammatory processes due to the chronic upregulation of proinflammatory cytokines, which are direct causes of respiratory allergies, rheumatoid arthritis, fibromyalgia, obesity, metabolic syndrome, type 2 diabetes, cancer and cardiovascular diseases. In addition, depression, insomnia, and chronic fatigue syndrome are caused by a reduction in cortisol levels [21]. Such diseases are the result of a continuous process of multidirectional interactions among the frontal lobe of the brain (which perceives stress), the autonomic nervous system, the endocrine system and the immune system [18]. A better understanding of the molecular actions of cortisol in the processes of memory and learning or in sleep disorders such as insomnia would facilitate progress in the prevention and both pharmacological and psychological treatment of stress disorders for those who are predisposed to such conditions [22]. Figure 1 shows the impact of stress on the psychoneuroimmunoendocrine axis.

## Relationship of epigenetic factors with psychoneuroimmunoendocrine factors

Epigenetics is the study of all non-genetic factors that interfere in the determination of ontogeny or in the development of an organism from fertilization to senescence. Such factors are involved in the heritable regulation of gene expression via methylation, acetylation and phosphorylation of DNA rather than alteration of the nucleotide sequence. The concept of epigenetics was coined by Conrad Waddington in 1953 and gained importance after the human genome project in 2003 [23, 24]. Living in urban areas, resulting in greater exposure to chemicals, reduced green spaces, and the consequent limited presence of flora, fauna and microbial life, is associated with immune dysfunction in humans. Reduced contact with nature and environmental microbiota appears to be related to a range of diseases including allergy and type 1 diabetes [25, 26]. Alterations in intestinal flora influence the development of not only asthma and allergies but also other chronic and recurring inflammatory disorders, such as type 1 diabetes, inflammatory bowel disease, obesity, and even psychiatric disorders [27]. Epigenetics has transformed our understanding of the impact of the environment on our genes and health, which in turn will potentially streamline many lines of research in psychoneuroimmunology seeking to explain how environmental cues are transduced into the genome [28, 29]. In turn, the psychosocial environment can substantially change behavior and alter nervous, endocrine and immune functions.

## Allergic diseases and the psychoneuroimmunoendocrine axis

A close relationship between allergic diseases and psychoneuroimmunoendocrinology exists [10, 30]. Stress negatively impacts patient quality of life, leading to development of depression, anxiety and unhealthy lifestyles along with secondary problems such as overweight and obesity, which negatively impact the control of atopic diseases [6]. Studies support this association and urge further investigation. A study of adolescents in the United States documented that atopic diseases such as asthma, allergic rhinitis and atopic dermatitis correlate with increased risk of anxiety and depression and that this risk is further increased when asthma and allergic rhinitis are concomitantly present [31]. Further research found that depressive disorders are common in individuals with asthma but that there is no correlation between the severity of asthma and the severity of depression [32]. The quality of life of adolescent patients with atopic dermatitis and underlying psychiatric disorders was also assessed: a high prevalence of anxiety and depression was found in these individuals. Studies have shown a link between quality of life and sleep loss and depression induced by atopic dermatitis [33]. Recently, the association between allergic rhinitis and psychiatric diseases such as depression and anxiety has been determined. In nine out of 11 studies, there was an association between allergic rhinitis and anxiety, and in ten out of 12 studies, there was an association of depressive disorder with allergic rhinitis [34]. Patients with attention deficit/hyperactivity disorder have a higher incidence of asthma, allergic rhinitis and atopic dermatitis than the general population. Children with atopic diseases are exposed to higher levels of inflammatory cytokines that are released due to an allergic response, and these cytokines can cross the blood–brain barrier and activate neuroimmunological mechanisms involved in emotions and behavior. Furthermore, activation in regions of the prefrontal cortex, potentially due to exaggerated and sustained release of inflammatory mediators, has been found. Another possible hypothesis explaining the relationship between these two diseases is based on the finding that allergic rhinitis is often associated with sleep disturbance, which may cause symptoms of daytime fatigue, inattention, irritability and impulsivity, which are in turn components of clinical attention deficit/hyperactivity disorder and its associated pathologies [35]. Urticaria occurs frequently in patients with psychiatric problems and emotional distress. Staubach P et al., found that 48 percent of patients with chronic spontaneous urticaria have at least one mental disorder; anxiety was the primary associated pathology, but depression and somatization disorders were also found [33, 36].

There is a relationship between obesity and allergic diseases. Adipokines, which are fat proteins that function as cytokines, chemokines and cytokine receptors have an important role in that relationship. At present, most studies on obesity, allergic diseases and asthma are based on the inflammatory and metabolic roles of leptin and adiponectin. Adiponectin is an anti-inflammatory protein that inhibits IL6, the transcription factor NFκB and TNF-α and that increases the concentration of IL1 and IL10. Adiponectin levels are decreased in obese people due to necrosis of fat tissue resulting from hypoxia, which causes infiltration of polymorphonuclear cells and macrophages that secrete IL6 and TNF-α and inhibit the synthesis of adiponectin [37]. Lectin is a proinflammatory protein that promotes the release of IL6 and TNF-α, decreases the activity of regulatory T cells, promotes Th1 lymphocyte activity and increases the levels of IFN-γ [38, 39].

There is a strong positive association between asthma incidence and lectin levels in prepubescent males and postmenopausal women. Obesity reduces progesterone levels in women, which lowers the levels of β2 adrenergic receptors, decreasing the relaxation of muscle in the respiratory tract [40]. The concentrations of both total and specific IgE in children and adolescents with allergic symptoms are higher among those who are overweight or obese [41].

Vitamin D deficiency is more common in the obese population, in whom there is an inverse relationship between serum vitamin D levels and the degree of obesity. Vitamin D has also been shown to skew T cells toward a less inflammatory state. For instance, 1,25(OH)2D3 decreases T cell-mediated IFN-γ production while increasing IL4 production [42, 43]. Both the generation and immunosuppressive capacity of Foxp3 + CD4+ regulatory T cells are increased by 1,25(OH)2D3 [44]. Moreover, recent studies showed that production of the inflammatory cytokine IL17 by T cells is prevented by 1,25(OH)2D3 [43, 45]. In line with these results, other groups have documented that the development of Th17 cells is negatively modulated by 1,25(OH)2D3 [46]. Production of IL21, IL22 and IL17 is also inhibited by physiologically relevant doses of 1,25(OH)2D3 in Th17-skewed T cells; this evidence suggests that principal changes in transcription are driven by the vitamin D receptor-transcription factor complex [43].

At the same time, vitamin D deficiency is associated with a decrease in immune cell proliferation as well as synthesis of cytokines, including IL1, IL2, IL6 and IL12, TNF-α and IFN-γ. These cytokines, which are upregulated in patients with obesity and metabolic syndrome, decrease the serum concentrations of vitamin D. Thus, it has been presumed that in overweight patients, as the amount of visceral adipose tissue increases, the kidnapping of vitamin D by adipose tissue increases. Secondarily, it is proposed that vitamin D deficiency or insufficiency is responsible for insulin resistance and thereby promotes metabolic syndrome [47]. Vitamin D deficiency has been associated with increased airway hyperresponsiveness, decreased lung function, reduced asthma control, and resistance to steroids [48]. A recently conducted study of treated asthmatic children showed that 84.2% of children with asthma had low levels of vitamin D. In that study, overweight was an important risk factor for vitamin D deficiency and insufficiency [49]. Another study found that vitamin D deficiency is associated with an increased risk of severe asthma in asthmatic adults (odds ratio [OR], 5.04; 95% confidence interval [CI]: 1.23 to 20.72; *p* = 0.02) and that high levels of vitamin D are related to a lower risk of hospitalization or emergency department visitation in the past year (OR, 0.90; 95% CI, 0.84 to 0.98; *p* = 0.04) [50]. Obesity and overweight have also been associated with increased residual capacity and increased risk of asthma. It has been observed that children with asthma are at an increased risk of exacerbations as well as uncontrolled asthma [51, 52]. It has also been shown that there is an association between low vitamin D levels, physical inactivity and high BMI [53]. Alternatively, vitamin D may reduce asthma severity and improve asthma control [54].

## The psychoneuroimmunoendocrine axis and its relationship to other relevant diseases

Based on the pathophysiological mechanisms described above, the psychoneuroimmunoendocrine axis has been related to neuropsychiatric diseases such as depression [4, 55–57] and schizophrenia [56, 58–61], metabolic syndrome [62, 63], rheumatologic and autoimmune diseases [64–72], irritable bowel syndrome [73, 74], periodontal disease [75, 76] and neoplastic diseases [62, 77], and these relationships warrant significant attention. Psychoneuroimmunology represents the challenge of health professionals to achieve multidisciplinary management of each of these pathologies.

## Psychoneuroimmunology and the naturalistic model

We propose a new medical model that has been described to be based on the concept of holistic medicine, in which biological, psychological, social and environmental aspects of the health-disease process are taken into account in the recommendation of a revised lifestyle. Through allostasis, the autonomic nervous system, the hypothalamic-pituitary-adrenal axis, the cardiovascular system, the immune system, the endocrine system and metabolism protect the body by preparing these systems to address both internal and external stress. This concept of allostasis complements the concept of stress. Allostasis represents the active adaptation process involving the production of mediators such as adrenal steroids, catecholamines, cytokines, neurotransmitters and other factors. After suffering chronic stress, adaptation responses or allostatic responses are initiated in the body. Inadequate or excessive responses following repeated stressful situations lead to allostatic load, which is the “price paid by the organism” for being forced to adapt to psychosocial or physical adversity. Thus, allostatic load constitutes the cumulative wear and tear resulting from chronic hyperactivity as an adaptation to the constant demands of life. The response to stress is physical, mental and behavioral and depends on basic personality as well as social, cultural, environmental and genetic factors. A new medical paradigm of health promotion and disease prevention is very important, as this paradigm supports lifestyle changes that increase resilience to stress and augment immune system defenses [78].

## Psychoneuroimmunology, education and stress management strategies

Health education must consider the need to educate people regarding their potential and shortcomings in assuming their own identity. Another contribution of health education is to orient people regarding the management of emotions in order to facilitate the appropriate channeling and expression of emotions, which is a form of disease prevention and, consequently, a reflection of health and wellbeing.

The discipline of health education has the major challenge of establishing principles and methodologies that enable people to learn healthy practices and lifestyles so as to enhance their capacity for resilience. In addition, health education seeks to develop and promote the process of addressing struggles or mishaps of life and of resisting, overcoming and transforming adversity in order to emerge strengthened or even renewed. The development of fundamental strategies for the prevention of disease and the recovery of health through health education interventions results in positive adaptation in contexts of great adversity. In addition, health education interventions should help people learn to take measures that enhance their ability to combat disease and that properly harmonize and balance mind-body function. Health promotion strategies should be directed toward prevention and resolution of health problems and toward improving quality of life [79].

As mentioned above, lifestyle changes that increase resilience to stress and enhance immune system defenses are indispensable. Adequate daily rest; a diet that decreases oxidative stress, including daily consumption of fruits, vegetables, legumes, essential fatty acids and trace elements; and physical exercises that activate the immune system, such as breathing exercises that increase breathing capacity, and elimination of cigarette, drug and alcohol use are among the lifestyle changes to be considered. In addition, focusing on psychological aspects such as tracing life goals, being flexible, maintaining harmonious communication with others, having a consistent attitude in life, optimism and proper management of emotions, can also help with stress management [80].

Diet appears to play an important role in stress management. Relations of multivitamins and minerals with stress have been described: the main identified antistress drugs contain vitamins E, B_1_, B_2_, B_3_, B_5_, B_6_, B_12_, and C, folic acid and the minerals zinc and iron [81]. Omega-3 fatty acids are very important for the functioning of the human brain. Poor intake of these acids induces several alterations in neurotransmission that can cause diverse psychiatric disorders, including schizophrenia and major depression. It has been observed that patients with psychiatric disorders who use fatty acid supplements exhibit a significant improvement in their symptoms. In addition, omega-3 fatty acids have been shown to be useful in decreasing antisocial behavior, hostility and aggressiveness in patients who are exposed to a psychologically stressful environment. Therefore, supplements containing omega-3 fatty acids can reduce such behaviors [82].

Exercise can be an effective stress management strategy and should be recommended for addressing acute, episodic acute, and chronic stress. One advantage of incorporating exercise with other stress management techniques is the psychological and physical beneficial effects of exercise. However, it is important to remember that exercise is only one component of a stress management program. Even though exercise may be effective in helping a person feel calmer, this change will not resolve the main triggers of chronic stress. It may be necessary to refer people suffering from chronic stress to professionals who can help them cope with their stressors [83]. Research on exercise and stress has typically focused on aerobic exercise. For instance, it has been reported that patients feel calmer after 20 to 30 min of aerobic exercise and that the calming effect of exercise can last for several hours afterwards. Recently, there has been an increase in the amount of research examining the role of body-mind types of exercise, such as yoga and Tai Chi in reducing stress. Nevertheless, there is limited research on the role of resistance exercise in managing stress [83]. Studies of humans and animal models have shown that being physically active improves the ability of the body to handle stress due to changes in hormonal responses and that exercise results in actions of brain neurotransmitters, such as dopamine and serotonin, that affect the body, state of mind and behavior. Additionally, exercise may serve as a time away or release from stressors. In a study of women attending a university who reported that studying was their main stressor, performing a constant exercise activity without performing a study activity and resting while exercising had a greater calming effect than quiet rest [84]. Recent publications on yoga or Tai Chi indicate that these types of mental exercise can be effective in reducing stress. Authors have suggested that the results should be viewed with caution because the quality of the studies varied [85, 86]. The decrease in stress reported in one review was similar to or greater than the reduction in other types of commonly used stress management strategies [87]. Lack of time is the limitation to performing exercise most commonly expressed by individuals. Lack of motivation, tiredness, and poor sleep and eating habits are additional factors associated with stress that can negatively affect compliance with an exercise regime [88].

Acupuncture may be effective in the treatment of chronic stress symptoms [89]. The main investigative findings concerning the effects of acupuncture on stress are outlined in Table 2.

**Table 2:** Acupuncture as a stress management strategy: randomized controlled trials

Wu, Y, Yuan, J, Feng, X. (2011) [141]	Acupuncture as an adjunct to anesthesia was found to help maintain stable hemodynamics and reduce stress responses during laparoscopic cholecystectomy surgery.
Kwong E, Yiu E. A. (2010) [142]	Acupuncture did not reduce salivary cortisol concentrations (and therefore could not reduce emotional stress) in female patients with dysphonia.
Middlekauff H, Hui K, Yu J. (2002) [143]	Acute acupuncture appeared to control excessive sympathetic arousal during mental stress in individuals with advanced heart failure.
Balk J, Catov J, Horn B. (2009) [144]	Acupuncture is associated with reduced stress during embryo transfer and increased pregnancy rates in women receiving in vitro fertilization.
Hui K, Marina O, Liu J et al. (2010) [145]	Acupuncture affects areas of the brain known to reduce sensitivity to pain and stress, promotes relaxation, and deactivates the analytical brain, which is responsible for anxiety.
Erickson K, Voss M, Prakash R et al. (2011) [146] Kim H, Park H, Shim H et al. (2011) [147]	Acupuncture improves stress-induced memory impairment and increases acetylcholinesterase reactivity in the hippocampus.
Park H, Kim H, Hahm D. (2010) [148]	Acupuncture reduces serum levels of corticosterone and the number of tyrosine hydroxylase-immunoreactive cells.
Lee A, Fan L. (2009) [149] Cheng K. (2009) [150]	Acupuncture regulates levels of neurotransmitters and hormones such as serotonin, noradrenaline, dopamine, neuropeptide Y and ACTH, thus altering the mood chemistry of the brain to help combat negative affective states.
Arranz L, Guayerbas N, Siboni L et al. (2007) [151]	Acupuncture reverses pathological changes in levels of inflammatory cytokines that are associated with stress reactions.
Kavoussi B, Ross B. (2007) [152]	Acupuncture reduces inflammation by promoting the release of vascular and immunomodulatory factors.

Mindfulness, a new therapeutic model proposed for the management of chronic stress, consists of an individual’s awareness of and attention to his or her symptoms of emotional distress experienced under chronic stress. This strategy facilitates therapies and enables the modifications necessary to improve lifestyle. This intervention is practiced by Manolete S. Moscoso at the University of South Florida. The purpose of this therapeutic intervention program is to instruct the individual in the relaxation response, reduce the level of chronic stress and change patterns of self-destructive behavior, obtaining an immune and neuroendocrine benefit that promotes the restoration of health and helps control the symptoms caused by medical treatments, which allows patients experiencing severe depression to counter the recurrence of this disease. Mindfulness allows focus and consciousness in the body through breathing, in the mind through thought, and the environment through the senses. The belief of “living on the run” from stress and emotional pain through daily life experiences contributes to fear, tension, anxiety, worry, anger and hostility. When behavior is modified in response to the difficulties of life and when it is understood that pain and pleasure are genuinely human experiences, an individual can achieve an adequate level of acceptance and peace [18, 62].

## Conclusions

Diseases are the result of an alteration at the bio-psycho-social level that can indicate lifestyle changes that should be made in addition to appropriate medical management and treatment. Emotions and stress significantly affect health and one’s susceptibility to a pathology, as well as one’s ability to recover from an illness. Psychoneuroimmunology should provide knowledge about the biological dynamics of conventional and alternative medicines for fighting disease. The psychoneuroimmunological axis comprises several disease-producing mechanisms in which different disciplines of medicine interact, implying the need for an integrative approach. The science of psychoneuroimmunology must go hand in hand with health education and the promotion of healthy lifestyles in order to attain patient health.

## Abbreviations

CNS: Central nervous system
CPR: C-reactive protein
IFN-γ: Interferon-gamma
IL: Interleukin
NK: Natural killer
TNF-α: Tumor necrosis factor-alpha
